# Distinctive Convergence Eye Movements in an Acquired Neurosensory Dysfunction

**DOI:** 10.3389/fneur.2020.00469

**Published:** 2020-06-16

**Authors:** Carey D. Balaban, Mikhaylo Szczupak, Alexander Kiderman, Bonnie E. Levin, Michael E. Hoffer

**Affiliations:** ^1^Department of Otolaryngology, University of Pittsburgh, Pittsburgh, PA, United States; ^2^Department of Neurobiology, University of Pittsburgh, Pittsburgh, PA, United States; ^3^Department of Communication Sciences & Disorders, University of Pittsburgh, Pittsburgh, PA, United States; ^4^Department of Bioengineering, University of Pittsburgh, Pittsburgh, PA, United States; ^5^Department of Otolaryngology, University of Miami Miller School of Medicine, Miami, FL, United States; ^6^Neurolign USA LLC, a subsidiary of Neurolign Technologies Inc. (formerly Neuro Kinetics, Inc.), Pittsburgh, PA, United States; ^7^Department of Neurology University of Miami Miller School of Medicine, Miami, FL, United States; ^8^Department of Neurological Surgery, University of Miami Miller School of Medicine, Miami, FL, United States

**Keywords:** vergence eye movements, pupil regulation, human, objective diagnosis, video-oculography

## Abstract

In late 2016, diplomats in Havana, Cuba, began presenting with a unique symptom complex after perceiving a strange noise and/or feeling a pressure field in their domicile. This report is a retrospective, quantitative analysis of video-oculography data of pupillary light reflex performance and binocular disparity-driven eye and pupil movements during the acute time period after the reported exposure. The patterns of response in these 19 individuals are markedly different than those seen in a group of individuals with the usual acute mild traumatic brain injury (17 subjects) and from 62 control subjects (21–60 years old) with no injury. Non-linear least squares regression was used to estimate the model parameters from the eye movement and the pupil measurements (1). Linear discriminant analysis was then used to identify a classifier for an objective discrimination of the groups with >91% accuracy and no confusion between the acute neurosensory findings among the members of the Havana diplomatic community and the subjects with acute mild traumatic brain injury. This pattern difference in eye and pupil behavior may be a useful screen to help objectively distinguish blunt trauma from Havana-type effects in the future and to guide the affected individuals to appropriate care.

## Introduction

Complaints of sudden-onset tinnitus, ear pain, and dizziness emerged in late 2016 to early 2017 among diplomatic personnel and their families in Havana, Cuba. These affected individuals often reported hearing a loud, high-frequency, localized sound, and were under the impression that it could follow them in a room. Examinations and formal testing at the University of Miami in the acute to the subacute time frames found had objective evidence of both an otolithic abnormality and of cognitive dysfunction in a group of 25 individuals (2). When 21 exposed individuals were examined in a more chronic time frame (average of 201 days post-perception of exposure), 20 subjects reported persistent symptoms and signs that resembled the aspects of mild traumatic brain injury (3). As noted previously (2), their findings are not inconsistent with either a partially compensated vestibulopathy or a mild brain trauma (or both). When seen at the University of Miami, these patients underwent diagnostic examinations that were guided by their symptoms and standard of care. As part of these examinations, all individuals underwent detailed video recordings of eye movements. These findings were reviewed, respectively, on an approved protocol (2) and formed the basis of the data set used in this study. The goal of this study was to provide a more detailed look at the eye motion seen in this patient group and compare these findings to other groups of individuals undergoing the same tests. The findings might allow investigators to begin to develop a set of objective findings that are typical for the acute neurosensory dysfunction among some members of the Havana diplomatic community.

The singular emergence of this cluster of individuals, the limited acute clinical information, the wide publicity about perceptions and symptoms, and the lack of etiologic information raise the practical issue of how one recognizes a similar presentation objectively in other individuals reporting the same perceptions. In the course of the acute examinations of the affected individuals from Havana, digital video records of eye movements were recorded for the qualitative assessment of pupillary light responses and vergence eye movements. These archival eye recording data have been analyzed retrospectively based upon algorithms from a recent publication that assessed both the vergence eye movements and the coordinated changes in pupil area during binocular disparity-driven convergence in more than 50 control subjects (1). Because convergence insufficiency (4–6) and pupillary light reflex effects (7) are described in the mild traumatic brain injury (mTBI) literature, detailed analyses are likely germane to the differential diagnosis of the Havana individuals. The patterns observed in this group of patients are compared with similar data from individuals with acute mTBI and our retrospective control group produced a discriminant function to differentiate among Havana affected, control, and acute mTBI samples. We hypothesize that the acute neurosensory presentation of these individuals will show features that are distinct from those of the standard acute mTBI.

## Materials and Methods

### Participants and Study Design

The analysis of de-identified, retrospective data was approved by the University of Pittsburgh Institutional Review Board and the institutional review board (IRB) at the University of Miami and the Madigan Army Medical Center. The de-identified data were analyzed from three cohorts of subjects:

#### Control Subjects

A cohort of 64 normal subjects came from the University of Miami and the Madigan Army Medical Center. Fifty-two subjects are described in a previous communication (1), supplemented by 12 subjects to span the age range of the mTBI and Havana affected cohorts. They provided data with written informed consent under protocols approved by the IRBs at the University of Miami and the Madigan Army Medical Center and in accordance with the Declaration of Helsinki. The subjects were 42 males and 22 females, ranging from 21 to 60 years of age (mean 32.3 ± 10.0 years of age, SD).

#### Mild Traumatic Brain Injury Subjects

A cohort of 18 subjects from the University of Miami, the Naval Medical Center San Diego, and the Madigan Army Medical Center (17 with complete data) provided data with informed consent under protocols approved by the IRBs at the University of Miami, the Naval Medical Center San Diego, and the Madigan Army Medical Center and in accordance with the Declaration of Helsinki. The 14 male and four female subjects, ranging in age from 20 to 43 years (mean 29.1 ± 8.1 years of age, SD), were diagnosed as having mTBI by both an emergency room physician as well as one of the authors (MEH). The criteria used for the diagnosis included a standard military definition of having a head injury, suffering an alteration or loss of consciousness, and having new neurosensory symptoms with onset at the time of injury and persisting until during our team's evaluation (all individuals ended up to have symptoms for at least 1 week). In addition, these individuals could not have been diagnosed with more than mild brain injury [e.g., no loss of consciousness (LOC) or LOC <30 min, no subdural hematoma, no need for admission to the intensive care unit, and a Glasgow Coma Score of 14 or above]. All individuals were evaluated in the acute period [tested 58 ± 36 h (SD) after injury]. In addition, these individuals had no significant medical issues, no history of ear disease or ear surgery, no history of developmental learning issues, and no history of concussion for at least 1 year, with no lingering sequelae before the current injury.

#### Havana Affected Subjects

There were 19 subjects with complete vergence and pupil test data among the 25 affected subjects described in Hoffer et al. (2). The entire cohort was composed of 15 males and 10 females (mean 43.2 ± 12.6 years of age, SD; all under 64 years of age). There were 23 subjects with pupillary light reflex tests (mean age 42.0 ± 7.6 years, SD) and 19 subjects who also had both binocular step and smooth pursuit vergence tests (mean age 40.5 ± 9.1 years, SD; under 56 years of age). The analysis of de-identified clinical video-oculographic data was approved by institutional review board of the University of Miami and the University of Pittsburgh, respectively.

### Pupillary Light Response and Binocular Disparity Vergence

Pupillary light responses and binocular disparity vergence eye movement performance were tested quantitatively with a clinical eye tracking system within a portable 3D head-mounted display system D × 100 [Neurolign USA, LLC a subsidiary of Neurolign Technologies (formerly Neuro Kinetics I-Portal, I-PAS), Pittsburgh, PA, USA]. Each eye views an independent circular portion of a 1,920 × 1,080-pixel stimulus display that subtends a 60° diagonal field of view. The subjects can adjust the focus of the video image for each eye across a ±4 diopter range. Video-based eye tracking is performed under continuous 940-nm infrared illumination at a sampling rate of 100 Hz. The pupils are identified by characteristic luminance boundaries. The pupil area is measured from each image at a resolution of 139 pixels/mm^2^. The instantaneous eye position is calculated from the centroid of the identified pupil area over a horizontal range of ±30° and a vertical range of ±20°.

Neurolign VEST software was used for testing and data collection. All stimuli were rendered in the virtual environment that was created by the enclosed video display, with synchronization of the stimulus refresh rates and the eye tracking sampling rate. Eye movement recordings were calibrated for a series of conjugate horizontal and vertical gaze shifts using spot targets subtending ~0.1° of the visual angle. Vergence angle is represented in degrees relative to zero at initial fixation. Normal consensual pupil responses were confirmed during the neurological examination. For testing the pupillary light reflex quantitatively, the subjects viewed binocularly a 5° (visual angle) disc centered on the visible area of each screen half while 13 homogeneous illumination flashes (0.42 to 65.4 cd/m^2^, 0.300 s on, 2.00–4.00 s off) were presented binocularly in an ascending order of intensity (**Figure 2**). Because the light-evoked and the vergence-related pupil movements were symmetric, the average area of the left and the right pupils was used in the final analyses.

Targets for the disparity fusion (“vergence tracking”) task were a white square with a red center that subtended ~0.1° of the visual angle for each eye. The total field luminance during the presentation of the square, measured with a spot luminance detector incorporating a LDM-9901 sensor (Gigahertz-Optik, Germany), ranged from 0.05 to 0.06 cd/m^2^. The vergence disparity step task began with the illuminated targets at a central fixation position for each eye. The targets were then shifted at 4-s intervals between a disparity requiring 1.5° convergence (from the initial fixation target) and a disparity requiring 1.5° divergence (from the initial fixation target) in order to achieve binocular fusion. Five cycles of alternating convergence and divergence were presented over a 40-s duration (**Figure 3**, right traces). By convention, convergence is represented as a positive vergence angle. For the vergence pursuit (tracking) task, the trial began with the illumination of the two monocular targets at the initial focal point phoria (equivalent to ~1 m in virtual depth). The target then moved smoothly through three cycles of a sinusoidal profile, such that the monocular targets moved simultaneously laterally and then medially to produce binocular disparity (i.e., the left eye target moved leftward, while the right eye target moved rightward, and then the left eye target moved rightward, while the right eye target moved leftward) with a cycle duration of 10 s (**Figure 3**, left traces). During this sinusoidal movement, the maximum deviation of the response from the initial position was ±2.6° of the visual angle in the horizontal plane.

### Data Analysis

In addition to the analysis of raw pupil area data, the pupillary light response range was used to normalize the pupil area for analysis of coordinated eye and pupil movements during the vergence tasks. For those analyses, the maximum (*A*_max_) and the minimum (*A*_min_) pupil areas were determined separately for the left and the right eyes for responses on the range of low-intensity (0.42 cd/m^2^) to high-intensity (65.4 cd/m^2^) stimuli. The range-normalized area (1) was calculated from raw data for an additional set of analyses .

The pupil data were analyzed both as peak constriction velocity for each flash intensity and from the parameter estimation for a model described in the Appendix. For the former observation, the pupil velocity was calculated from the pupil area data (in mm^2^) with a “fitted slope” method, which fits a straight line between points from i–*n* to i + *n* (*n* = half-width of a user-specified window. The numerator for the slope calculation is the sum (points multiplied by offset), where offset is the range from –*n* to *n*, while the denominator is *n* (*n* + 1) (2*n* + 1)/3. The product of the slope and the sampling frequency yields the velocity. The half-width of the window was five points (50 ms) at a sample rate of 100 Hz.

**Figure 1:**
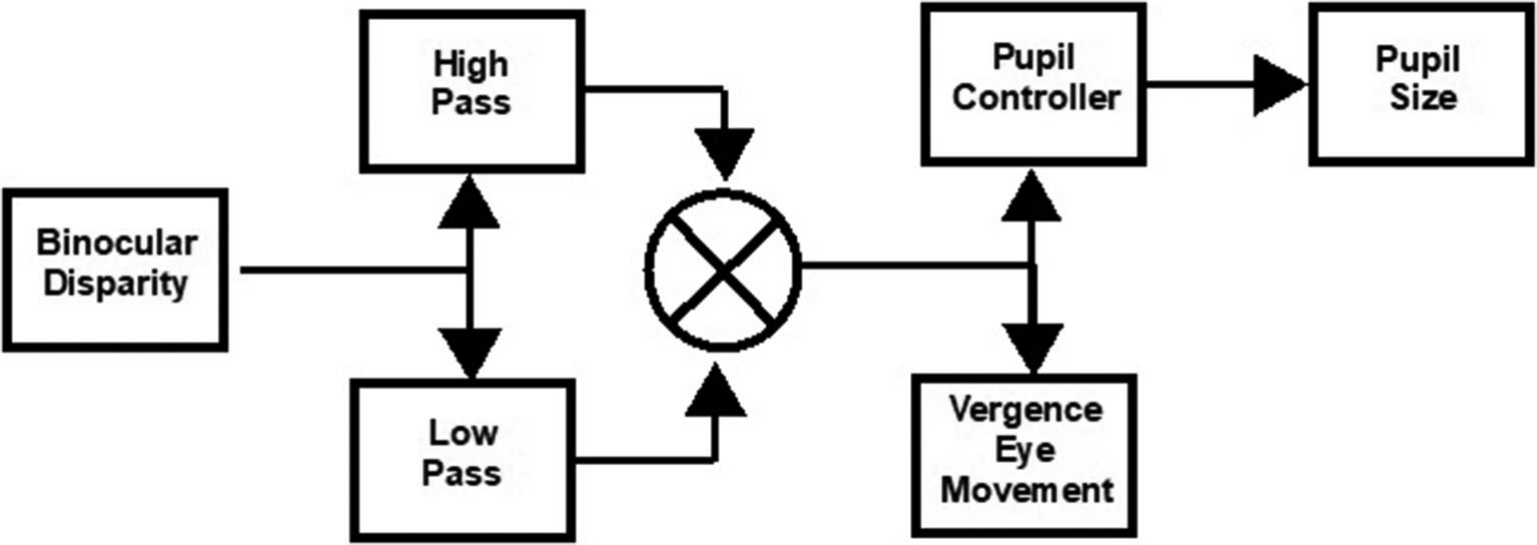
Schematic of approach for model estimation of binocular disparity-driven vergence eye movement and pupil responses (1). The transfer functions are described in the text. The pupil controller Laplace form was adopted from Sun et al. (8) as a parsimonious descriptor of the data.

The eye movements in the disparity step and the pursuit tasks were modeled as the weighted sum of first-order high- and low-pass representations of the vergence target position with a processing delay (1). The general architecture of this simple model is diagrammed in Figure 1. Data fits by this model to the vergence eye movement of control subjects had high average coefficients of determination, which were 0.82 ± 0.04 for the step disparity task and 0.91 ± 0.02 for the pursuit disparity task. They are used as an adequate parsimonious model, with fewer free parameters for estimation than the more elaborate models such as that of Maxwell et al. (9).

Non-linear least squares regression (“lsqnonlin.m” function in MATLAB) was used to estimate the parameters for the vergence disparity response as a weighted sum of high-pass ($\frac{{\text{K}}_{\text{vh}}}{\text{s}}{\text{e}}^{-{\text{t}}_{\text{v}}\text{s}}s+1$) and low-pass (${\frac{{\text{K}}_{\text{vl}}}{\text{e}}}^{-{\text{t}}_{\text{v}}\text{s}}0.25s+1$) processes, with delay *t*_v_ and gains *K*_vh_ (phasic process) and *K*_vl_ (tonic process), respectively. The delay parameter accounts for the reaction time to the binocular disparity step stimulus; it was set at zero for the binocular disparity pursuit task. Based upon Sun et al. (8), the pupil controller dynamics were fitted from the vergence data by a transfer function for pupil motion, ${\frac{{\text{K}}_{\text{p}}}{\text{e}}}^{-{\text{t}}_{\text{p}}\text{s}}0.28s+1$, with delay *t*_p_ and gain *K*_p_, which estimates the near response sensitivity directly. As shown in our previous publication (1), the residuals from this approach showed no activity in the range of the vergence eye movements, which indicates the descriptive adequacy of this simple model for the present analyses. The symmetry of the convergence responses was tested by fitting separate gains for convergence vs. divergence and for pupil constriction vs. dilatation.

Statistical analyses were performed in SPSS version 24 (IBM Corporation, Armonk, NY, USA). Kolmogorov–Smirnov tests (Lilliefors correction) were used to test the assumption of a Gaussian distribution for each dependent variable and group. For variables that were not rejected as Gaussian, paired comparisons between group data were performed by analysis of variance, followed by Tukey's highest significant difference (HSD) and Games–Howell *post hoc* tests. When the Gaussian assumption was rejected (*p* < 0.05), a distribution-free Kruskal–Wallis approach in the SPSS Non-parametric tests → Independent Samples menu was used. Stepwise discriminant analysis for the control, acute mTBI, and Havana affected subjects was performed on the parameters describing vergence eye and pupil movements, with a Wilks-lambda criterion and a one-out validation.

## Results

**Figure 2:**
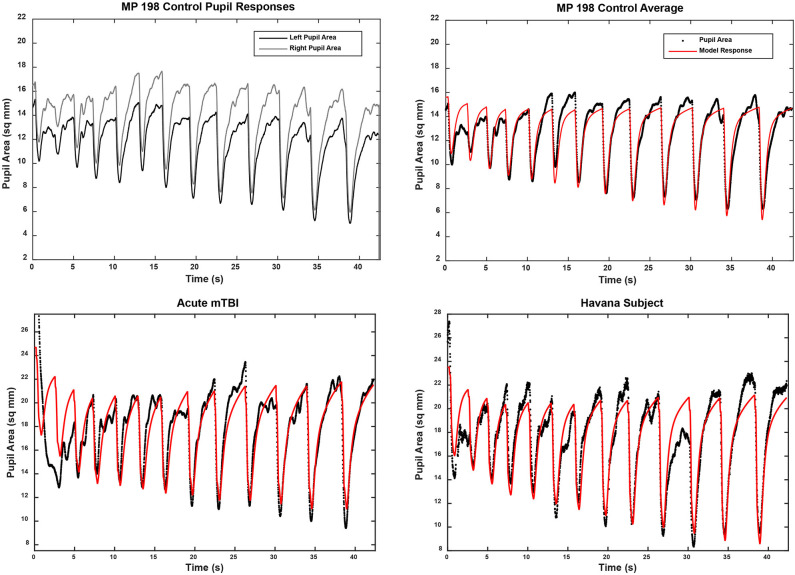
Pupil area responses during binocular presentation of 13 homogeneous illumination flashes (0.42 to 65.4 cd/m^2^, 0.310 s on, 2.03 s off). The upper left panel shows the parallel and the highly coherent responses of each eye from a control subject. In the other panels, the black traces show the average of the responses of both eyes from three subjects (one from each subject group). The modeled response for each trace is shown in red based upon the approach described in the supplemental material. Note the strong similarity in the dynamic responses across groups, which was confirmed by the lack of differences between the response parameters in quantitative analyses (see text).

The pupillary light response to a series of binocular light flashes is shown in Figure 2. All of the subjects displayed parallel right and left pupil size traces, which is illustrated for one control subject in the upper left panel (Figure 2). Hence, given the high coherence between the pupils, the main analyses were conducted on their averaged area. Note that a lumped parameter dynamic model (described in Appendix), based upon a series of published model iterations from the Stark laboratory (8, 10–13), provided a reasonably robust fit to the responses in control, acute mTBI, and Havana affected subjects. There were no significant group differences in any parameter of dynamic behavior. However, two significant differences emerged in parameters between groups (Table 1). First, the average pupil area was significantly smaller in the Havana affected group than that of the control subjects, with an intermediate baseline pupil area in the acute mTBI subjects. Because there is a significant negative linear relationship between the average baseline pupil area and age in the control subjects (area = −0.342 ^*^ age + 29.176, *r* = −0.499, *p* < 0.001), the analysis was repeated with age as a covariate, which eliminated significant group differences (Table 1). Second, the goodness of fit was significantly higher (mean *R*^2^ > 0.68) for both the control and the Havana affected individuals than in the acute mTBI group (mean *R*^2^ = 0.55). The goodness of fit was uncorrelated with age. A reduction in static pupil size in the affected Havana subjects during the light reflex testing was also observed for the average pupil area during the disparity step (control: 19.77 ± 0.92 mm^2^, mTBI: 16.14 ± 1.72 mm^2^, Havana affected: 13.35 ± 1.67 mm^2^; *p* < 0.01 vs. control, Kruskal–Wallis test) and the disparity pursuit (control: 17.63 ± 0.91 mm^2^, mTBI: 15.26 ± 1.70 mm^2^, Havana affected: 12.66 ± 1.65 mm^2^; *p* < 0.05 vs. control, Kruskal–Wallis test) responses. Because there are significant negative linear relationships between these measures and age in the control subjects, the analyses were repeated with age as a covariate (Table 1). The correction for age indicated that only the mTBI subjects had smaller average pupil areas than those of the controls.

**Table 1:** Parameter estimates for pupil light responses in the three groups of subjects (see text for details).

	**Control group**	**Acute mild traumatic brain injury (mTBI)**	**Havana affected**	**Tukey's highest significant difference or Kruskal–Wallis (*p* < 0.05) comparisons**	**Least significant difference (*p* < 0.05) comparisons**
**Unadjusted measures**					
Light reflex average baseline pupil area (mm^2^)	18.24 ± 0.83 mm^2^ (Gaussian)	16.46 ± 1.65 mm^2^ (Gaussian)	14.15 ± 1.37 mm^2^ (Gaussian)	C > HA; C = mTBI; HA = mTBI	
Light response fit to model (*R*^2^, coefficient of determination)	0.68 ± 0.02 (Gaussian rejected)	0.56 ± 0.04 (Gaussian)	0.74 ± 0.03 (Gaussian)	C = mTBI; C = HA; HA > mTBI	
**Age-adjusted measures for average pupil area in each task with significant age relationship (basis age: 33.3939 years)**					
Light reflex average baseline pupil area (mm^2^)	17.92 ± 0.78 mm^2^	14.62 ± 1.53 mm^2^	16.37 ± 1.50 mm^2^		NS for all
Disparity step average pupil area (mm^2^)	19.38 ± 0.83 mm^2^	14.51 ± 1.64 mm^2^	16.22 ± 1.62 mm^2^		C = HA; C > mTBI; HA = mTBI
Disparity pursuit average pupil area (mm^2^)	17.29 ± 0.84 mm^2^	13.62 ± 1.66 mm^2^	15.25 ± 1.64 mm^2^		C = HA; C > mTBI; HA = mTBI

For the analyses of the peak constriction velocity data (a measure of dynamic performance), the only significant effect was noted at one flash intensity (47.8 cd/mm^2^). For that stimulus, the response was reduced significantly in the acute mTBI group relative to the control group (HSD test, *p* < 0.05, with the Havana exposed subjects not differing from either group).

**Figure 3:**
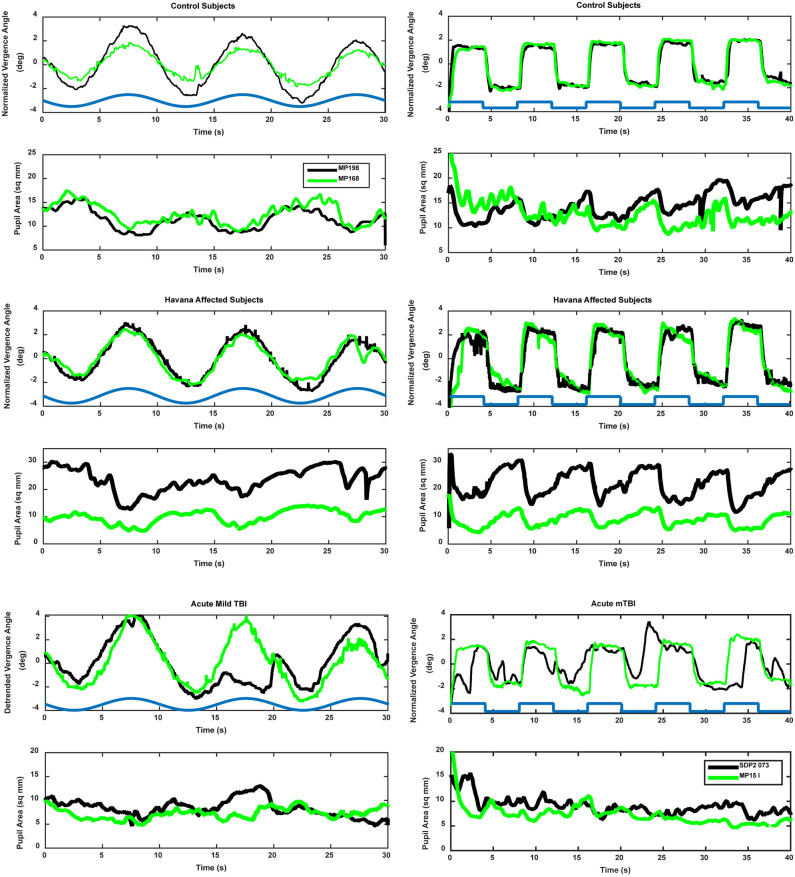
Examples of vergence (upper trace, convergence in the positive direction) and concurrent pupil area changes (lower trace, area in mm^2^) in control (upper panels), Havana affected (middle panels), and acute mild traumatic brain injury (lower panels). The time course of the retinal disparity stimulus is shown by the heavy blue line in the convergence panels, offset above the responses for illustrative purposes. Two different subjects (black and green traces) are shown for each group. The left panels show the responses during the binocular disparity pursuit task. The responses during the binocular disparity step task are shown on the right.

**Figure 4:**
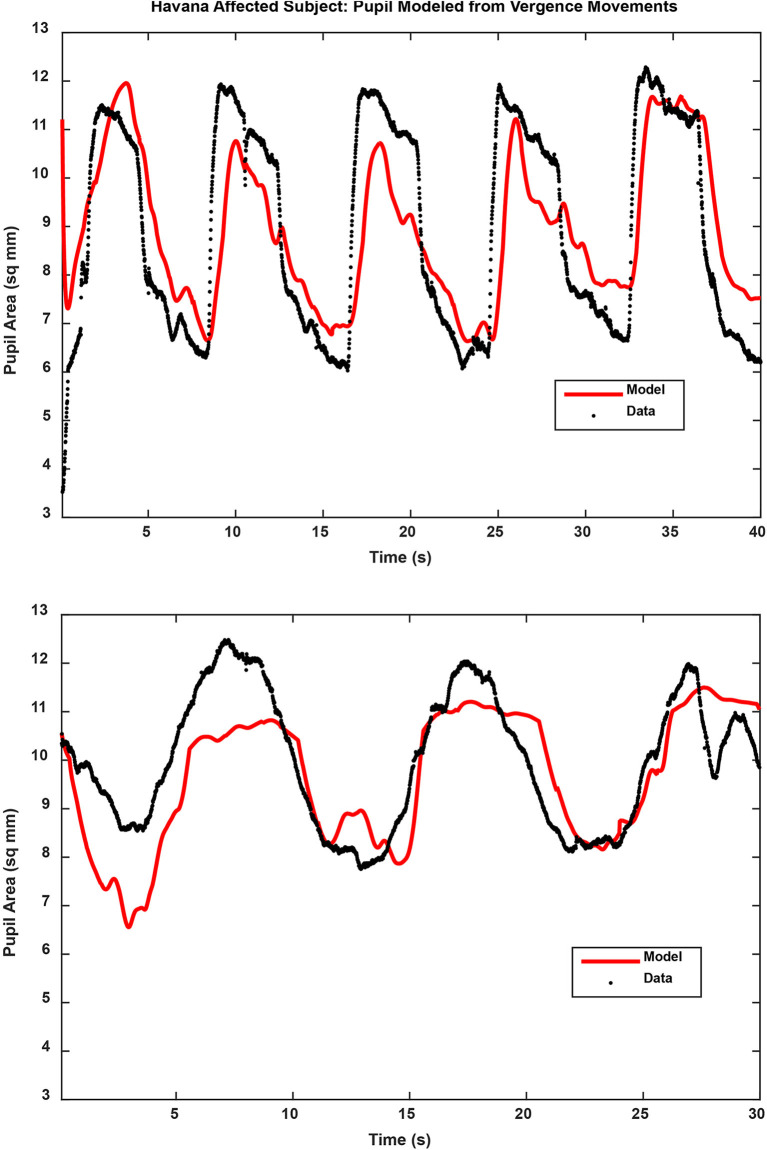
Pupil traces (black) are shown for the binocular disparity step response (upper panel) and binocular disparity pursuit response (lower panel) from a Havana affected subject. The model fit, based upon the eye movement vergence response, is shown for each trace in red. The full results of the analyses are described in the text.

Binocular disparity-driven tests provide a way to assess the coordination of movements related to convergence eye movements (1). Two test paradigms involve the presentation of small target spots to each eye to evoke convergent or divergent eye movements by moving toward the nose or away from the nose. For the disparity pursuit task, the targets move gradually to produce coordinated, sinusoidal convergence eye movements and changes in pupil area (Figure 3, left panels). Hence, it tests a low-frequency response. For the disparity step test (Figure 3, right panels), the targets move abruptly. It is included to examine the response at higher frequencies. In normal subjects, the dominant pattern of coordination is termed “near response”: pupil constriction (decreased pupil area) during convergence and pupil dilation (increased pupil area) during divergence. The behavior of normal subjects and the methods of analysis are described in detail in a recent publication (1). Examples of the fidelity of the fit of the model analysis to the pupil data (from the vergence eye movements) are shown in Figure 4 for one subject from the Havana affected group.

### Disparity Step Task: Vergence Eye Movements

The binocular disparity step response test revealed several differences between the control, acute mTBI, and Havana affected subject groups. The traces in Figure 3 show sharp, well-demarcated “square wave” vergence eye movement responses in the control and Havana affected groups, accompanied by robust pupillary responses. The eye movement and the pupil responses were attenuated and less sharply demarcated in the acute mTBI group.

The analysis of the responses (Table 2) showed that the acute mTBI group had significantly lower gains for the low-pass components of the eye movements than either the control or the Havana affected subjects (Kruskal–Wallis tests, *p* < 0.01) as well as a significantly poorer goodness of fit (HSD tests, *p* < 0.01). By contrast, the disparity step-driven eye movements in the Havana affected subjects did not differ significantly from those of the control subjects.

**Table 2:** Significant group differences in model parameter values for the disparity step responses.

	**Control group**	**Acute mild traumatic brain injury (mTBI)**	**Havana affected**	**Tukey's highest significant difference or Kruskal–Wallis (*p* < 0.05) comparisons**
Low pass convergence eye movement modulation depth (*K*_vl_ converge direction)	1.43 ± 0.09° (Gaussian rejected)	0.59 ± 0.17° (Gaussian rejected)	1.42 ± 0.16° (Gaussian rejected)	C > mTBI; C = HA; HA > mTBI
Low pass divergence eye movement modulation depth (*K*_vl_ diverge direction)	1.51 ± 0.08° (Gaussian rejected)	0.65 ± 0.16° (Gaussian rejected)	1.38 ± 0.15° (Gaussian rejected)	C > mTBI; C = HA; HA > mTBI
Vergence *R*^2^	0.82 ± 0.04 (Gaussian rejected)	0.48 ± 0.07 (Gaussian rejected)	0.67 ± 0.07 (Gaussian rejected)	C > mTBI; C = HA; HA = mTBI (*p* = 0.05)
Pupil delay re: vergence in ° (s) [re: % light response range per degree vergence]	0.16 ± 0.02 s (Gaussian rejected)	0.13 ± 0.04 s (Gaussian rejected)	0.01 ± 0.04 s (Gaussian rejected)	C = mTBI; HA < C; HA = mTBI
	[0.07 ± 0.02] (Gaussian rejected)	[0.09 ± 0.04] (Gaussian rejected)	[−0.08 ± 0.04] (Gaussian rejected)	[C = mTBI; HA < C; HA < mTBI]
Pupil constriction sensitivity in mm^2^/° (re: vergence)	2.10 ± 0.22 mm^2^/° (Gaussian)	0.76 ± 0.41 mm^2^/° (Gaussian rejected)	1.89 ± 0.38 mm^2^/° (Gaussian)	C > mTBI; HA = C; HA = mTBI
[% light response range per degree vergence]	[7.5 ± 1.0%/°] (Gaussian)	[5.3 ± 1.9%/°] (Gaussian)	[16.7 ± 1.8%/°] (Gaussian rejected)	[C = mTBI; HA > C; HA > mTBI]
Pupil (re: vergence) *R*^2^	0.47 ± 0.03 (Gaussian)	0.32 ± 0.05(Gaussian)	0.55 ± 0.05(Gaussian)	C = mTBI; HA = C; HA > mTBI

### Disparity Step Task: Dynamic Pupil Responses

A different picture emerged from the analyses of the dynamic pupil responses during the disparity step task. These movements are modeled as a parallel response to the signal generating the vergence eye movements [e.g., Balaban et al. (1)]. Separate analyses expressed the magnitude of the pupil response during disparity-driven vergence as either (1) pupil area per degree vergence (mm^2^/°) or (2) pupil area as a normalized percentage of light reflex range per degree vergence. Figure 3 (right panels) shows the distinct coordinated response of the pupil during the step vergence eye movements in two control and two Havana affected subjects and the much less distinct pupil responses in two subjects from the acute mTBI group. The reduced pupil coupling to the vergence eye movements in the latter group was apparent from the *R*^2^ values for the model fits, which indicated that the eye movement component explained an average of 47% of pupil variability in the control subjects and 55% of the variability in Havana affected subjects, but only 32% of the variance (HSD test, *p* > 0.01 re: either group) in the acute mTBI subjects.

Figure 4 shows the examples of model performance for a representative Havana affected subject. The Havana affected subjects showed a shorter estimated pupil response delay than the control group for mm^2^/° data (HSD test, *p* < 0.05; marginal for comparison with the mTBI group) and the mTBI group for data normalized to the pupil light response (HSD test, *p* < 0.05; marginal for comparison with the control group). The pupil area modulation of the Havana affected subjects, measured in mm^2^/°, did not differ from that of the control for either constriction or dilation. In the acute mTBI group though, these responses were altered significantly with rectification of the response in the dilation direction. Finally, for data expressed as % pupil range, the Havana affected subjects had more robust pupil constriction responses than either the control or the acute mTBI group (Kruskal–Wallis tests, *p* < 0.01). The response sensitivity in the acute mTBI group did not differ from that of the control subjects.

### Disparity Pursuit Task: Vergence Eye Movements

The binocular disparity pursuit task tests the disparity vergence performance over a slow, smooth pursuit cycle of 10 s. The traces in Figure 3 (left traces) show smooth, sinusoidal vergence eye movement tracking responses in the control and the Havana affected groups, with coordinated pupillary responses that display a clear sinusoidal component. The eye movement and the pupil responses were small and less distinct in the acute mTBI group.

The analyses of the disparity pursuit task data (Table 3) revealed reduced vergence eye movement modulation (low pass) in the acute mTBI group (Kruskal–Wallis test, *p* < 0.01) relative to that of the control group, with the Havana affected group showing a reduction so that it differed from neither the control subjects nor the acute mTBI subjects. As in the case of the disparity step task, the goodness of fit (*R*^2^) for vergence eye movements (to the stimulus profile) showed no difference between the control and the Havana affected subjects. Moreover, as in the case of the binocular disparity step task, the goodness of fit of the vergence eye movements of the acute mTBI subjects to the stimulus profile was reduced relative to those of both the control and Havana affected subjects (HSD tests, *p* < 0.01). These findings suggest that there is an impairment of the fidelity of disparity-driven vergence pursuit eye movement control in the acute mTBI subjects, but not in the Havana affected subjects.

**Table 3:** Significant group differences in model parameter values for the disparity pursuit responses.

	**Control group**	**Acute mild traumatic brain injury (mTBI)**	**Havana affected**	**Tukey's highest significant difference or Kruskal–Wallis (*p* < 0.05) comparisons**
Low pass convergence modulation depth (*K*_vl_ converge direction)	2.32 ± 0.10° (Gaussian)	1.71 ± 0.18° (Gaussian)	1.84± 0.16° (Gaussian rejected)	C > mTBI; C > HA; HA = mTBI
Low pass divergence modulation depth (*K*_vl_ diverge direction)	2.25 ± 0.09° (Gaussian)	1.75 ± 0.16° (Gaussian rejected)	1.87 ± 0.14° (Gaussian rejected)	C > mTBI; C = HA; HA = mTBI
Vergence *R*^2^	0.91 ± 0.02 (Gaussian rejected)	0.58 ± 0.04 (Gaussian rejected)	0.91 ± 0.04 (Gaussian rejected)	C > mTBI; C = HA; HA > mTBI
Pupil constriction gain in mm^2^/° (re: vergence)	1.67 ± 0.14 mm^2^/° (Gaussian rejected)	1.68 ± 0.217 mm^2^/° (Gaussian rejected)	1.46 ± 0.23 mm^2^/° (Gaussian)	NS in mm^2^/°
[% light response range/° vergence]	[7.4 ± 0.6%/°] (Gaussian rejected)	[5.9 ± 1.2%/°] (Gaussian rejected)	[11.8 ± 1.0%/°] (Gaussian)	[C = mTBI; HA > C; HA > mTBI]
Pupil dilation gain in mm^2^/° (re: vergence)	0.42 ± 0.14 mm^2^/° (Gaussian)	−0.61 ± 0.26 mm^2^/° (Gaussian rejected)	0.56 ± 0.22 mm^2^/° (Gaussian)	C > mTBI; C < HA; HA > mTBI
[% light response range/° vergence]	[8.3 ± 0.9%/°] (Gaussian rejected)	[7.2 ± 1.7%/°] (Gaussian)	[6.5 ± 1.5%/°] (Gaussian)	NS in %PLR/°
Pupil (re: vergence) *R*^2^	0.50 ± 0.02 (Gaussian)	0.30 ± 0.04 (Gaussian)	0.54 ± 0.03 (Gaussian)	C > mTBI; C = HA; HA > mTBI

### Disparity Pursuit Task: Dynamic Pupil Responses

As mentioned previously regarding Figure 3, the subject groups differed in the appearance of the components of the pupil area responses that were related to the vergence eye movements. The *R*^2^ values indicate that the eye movement component explained at least 50% of pupil variability in the control and the Havana affected groups, but only 32% of the variance (HSD test, *p* > 0.01 re: either group) in the acute mTBI subjects. The pupil area response (re: vergence eye movements) for constriction during the disparity pursuit task did not differ between groups when expressed as mm^2^/° (Table 3). However, when pupil area was normalized as a percentile of the pupil response range, the Havana affected subjects had significantly higher sensitivity per degree of eye movement than either the control or the acute mTBI subjects. The coordinated pupil response sensitivities (pupil area re: vergence angle) did not differ between the step and the pursuit tasks for any groups.

### Objective Classification of Subject Groups From Vergence and Pupil Responses

The linear discriminant analysis (Wilks-lambda criterion) demonstrated that the results of the two binocular disparity vergence and the pupillary light response tests are sufficient to classify individual subjects with high accuracy as control, acute mTBI, or Havana affected (Table 3). The data used in this analysis expressed the pupil responses as mm^2^/°. The canonical discriminant function for this three-way classification (Table 4) identified two classifier dimensions that produced a remarkable 91.8% correct rate overall, with a one-out cross-validation correct classification rate of 81.6%. Most notably, there were no classification errors between the Havana affected and the acute mTBI subjects. The three-way group separation remained at 89.8% correct overall (with no cross-classification errors between the mTBI and the Havana affected groups) when the variables with a significant age relationship were adjusted to a value for a standard age of 33.3939 years based upon estimated linear relationships in the control group.

**Table 4:** Discriminant function for control, acute traumatic brain injury (mTBI), and Havana affected patients from binocular disparity tests.

	**Control (predicted)**	**mTBI (predicted)**	**Havana affected (predicted)**
**Classification results (one-out in square brackets)**			
Control	61 [56]	1 [2]	0 [4]
mTBI	4 [6]	13 [11]	0 [0]
Havana affected	3 [6]	0 [0]	16 [13]
	**1**	**2**	
**Standardized canonical discriminant function coefficients**			
Vergence step modulation low-pass gain, converging direction	0.455	−0.651	
Vergence step modulation low-pass gain, diverging direction	0.674	0.320	
*R*^2^ for vergence step modulation	−0.764	0.495	
Pupil (re: vergence) step modulation in mm^2^/°, converging direction	−0.053	1.003	
Pupil (re: vergence) step modulation in mm^2^/°, diverging direction	0.142	−0.280	
*R*^2^ for pupil (re: vergence) step modulation	−0.178	0.215	
Pupil (re: vergence) step delay in % pupil range/°	−0.292	0.256	
Pupil (re: vergence) step gain, constriction direction in % pupil range/°	0.365	−0.835	
Mean pupil area (in mm^2^) for vergence step	0.149	1.430	
Vergence pursuit modulation low-pass gain, converging direction	0.079	0.275	
Vergence pursuit modulation low-pass gain, diverging direction	−0.580	−0.277	
*R*^2^ for vergence pursuit modulation	1.044	0.629	
Pupil gain (re: vergence pursuit), constriction direction in % pupil range/°	0.440	−0.193	
Pupil gain (re: vergence pursuit), dilation direction in % pupil range/°	0.096	0.596	
Mean pupil area during vergence pursuit (in mm^2^)	−0.162	−0.441	
Pupil area in mm^2^ (initial 200 ms of light response test)	0.165	−0.799	
*R*^2^ for model fit to pupillary light reflex test	0.415	−0.155	
Mean pupil baseline between flashes in pupillary light test (in mm^2^)	−0.237	−0.438	
Maximum pupil constriction velocity during pupillary light test	−0.299	−0.084	

*Stepwise discriminant analysis, Wilks–lambda criterion, Vergence test data only; the cross-validated estimates are in brackets. The raw data analyses were 91.8% correct overall; 81.6% correctly classified in one-out cross-validation. The three-way separation with age-adjusted data (based on a control sample regression relationship to a standard age of 33.3939 years) was very similar, 89.8% correct overall, with no cross-classification errors between the mTBI and the Havana affected groups*.

**Figure 5:**
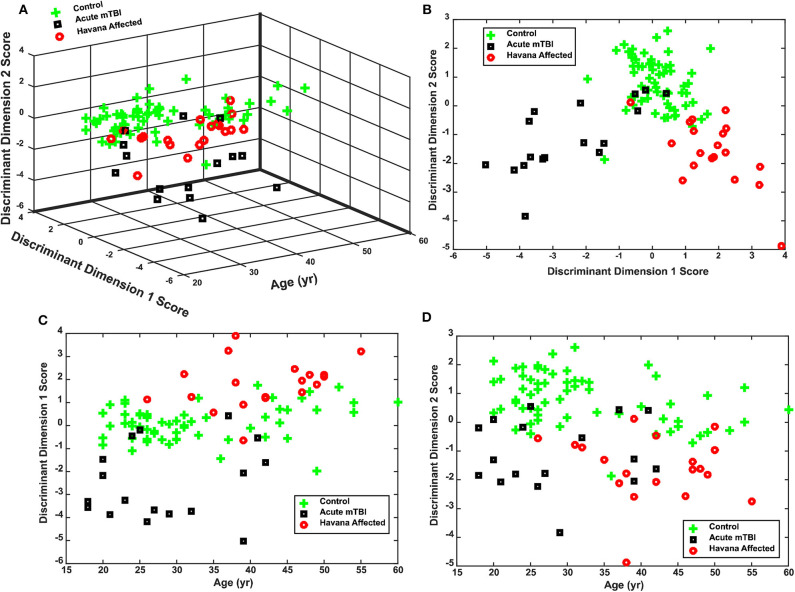
Performance of discriminant function for classifying control, acute mild traumatic brain injury (mTBI), and Havana affected subjects. **(A)** Three-dimensional plot of the distribution of individual discriminant function scores for subjects as a function of age at time of testing. Note that the group separation does not vary with age. **(B)** Plot of the relationship between the two discriminant dimensions, collapsed across subject ages. **(C)** Separation of acute mTBI, control, and Havana affected subject by discriminant dimension 1 across ages. **(D)** Separation of acute mTBI, control, and Havana affected subject by discriminant dimension 2 across ages.

The separation of the subject groups by the discriminant scores, across ages from 20 to 60 years, is shown in Figures 5A–**D**. The basic separation is shown for the scores in Figure 5B. Figures 5C,D show that the discriminant dimension scores were uncorrelated with the age of the subjects. Dimension 1 produced scores centered around zero for the control subjects, negative scores for the acute mTBI group, and positive scores for the Havana affected individuals (Figure 5B) across the entire age range of the subjects (Figure 5C). Dimension 2 showed lower scores for either the Havana affected or the acute mTBI group (Figures 5B,D). Thus, the distinctive group effects were consistent for the disparity step and the disparity pursuit tests.

## Discussion

In the time period since these symptom patterns were reported initially among the Havana diplomatic community, there has been a great deal of concern regarding the characterization of symptoms affecting the documented cases as well as the extent of the phenomenon in other populations at risk and in the general public. Dozens of individuals, both inside and outside the diplomatic community, have come forward with purported symptoms and, in the vast majority of cases, they have been diagnosed as not having a history and symptoms consistent with the group that we have seen from the Havana diplomatic community. We have termed this presentation as “worried well.” This fact alone, as well as the potential ongoing threat to diplomatic and other “forward” deployed assets (who face myriad other threats as part of their daily work), creates a critical need for standardized, accurate diagnostic criteria.

As we have discussed in our initial publication (2), this study is limited by being a retrospective analysis of the data obtained on this group of individuals in which only medically necessary information could be collected. In addition, the study is limited by a small sample size. Nevertheless, this patient population represents the *only* opportunity to report the presenting symptoms in any population seen acutely, without the influence of outside attention or a “pre-knowledge” of symptomatic complaints. Knowledge of the unbiased presenting symptom patterns is important since new cases as well as many cases of the worried well (worried but unaffected individuals) have been reported around the globe.

The current paper presents objective clinical findings that help to distinguish the truly affected individuals from normal subjects and from a cohort with acute mTBI. These metrics effectively classify these individuals from young adulthood through their early 60 s (the age range of our exposed subjects; see Figure 5), with no cross-classification errors between the Havana affected subjects and the acute mTBI subjects. We suggest that pupillary light reflex and binocular disparity vergence tests can be utilized to produce sensitive and selective measures that, paired with otolith tests (subjective visual vertical and vestibular-evoked myogenic potentials), identify symptomatic Havana-type exposures objectively on the basis of clinical features. These tests are ready for operational use to help distinguish the “worried well” and the symptomatic disorders with other potential causes.

To our knowledge, this report is the first demonstration of the potential diagnostic utility of a quantitative analysis of binocular disparity-driven eye and pupil movements. Although these movements are highly consistent in control subjects (1), the neurologic significance of the differences that we observed has yet to be probed. Hence, the features that distinguish the acute mTBI and the Havana Affected subjects from the control sample need to be viewed as objective, empirical “markers.” One feature of interest is that the rapid step and the slower smooth pursuit (0.1 Hz) binocular disparity tasks showed consistent results across the different subject groups. For example, the subjects with acute mTBI had lower magnitude vergence eye movements in either task than the control or the Havana affected subjects. The Havana affected subjects, on the other hand, had smaller baseline pupil areas than the control subjects for light reflex testing and during binocular disparity tests, associated with larger pupil changes per degree of vergence when data were normalized to the pupil area range.

Diagnosis of convergence insufficiency (e.g., in mTBI) is typically documented by (1) a minimum near point of convergence >6 cm from the face, (2) greater exodeviation for near objects than far objects, and (3) a positive fusional vergence during a prism test. These findings assess the final behavior of fusion on static targets. The measures utilized in this study assess the dynamic behavior of the eyes and the pupil during the responses to binocular disparity. The acute mTBI subjects showed a distinct pattern of changes in both binocular disparity tasks. Specifically, the magnitude of the eye movements was depressed significantly and symmetrically in both converging and diverging directions, accompanied by an effect on the magnitude of pupil responses (per degree vergence) for both constriction and dilation in the disparity step task and only dilation in the disparity pursuit task. These findings indicate that the amplitudes of the convergence movements were reduced significantly in the subjects with acute mTBI relative to the control subjects. For the step task, the magnitude was less than half the control value, which would be consistent with an apparent increase in the distance of the near point of convergence (relative to the subject's head). These dynamic movement findings are likely to be predominantly a supranuclear oculomotor control phenomenon that is related to generating the parallel drive to the extraocular muscles and the pupil controller (14).

Because the etiology of the consistent patterns of findings in these individuals is unknown, it is important to also consider the possibility of functional (or psychogenic) disease. Stated bluntly, one entertains a psychogenic or functional diagnosis after excluding known organic explanations. The appearance and the diagnosis of changes in pupillary size and reactivity are among the five psychogenic patterns that are discussed in neuro-ophthalmologic literature (15–19). However, it is noteworthy that the pattern of oculomotor and otologic findings in this cluster of individuals has no precedent in the “psychogenic” literature. Further, three arguments suggest that it is premature to invoke a functional etiology. Firstly, the new eye movement findings are sufficiently distinctive to reliably classify these individuals, control subjects, and subjects with acute mTBI with high accuracy (>91% by discriminant analysis). Secondly, the affected individuals reported perceptions that coincided with symptom onset. Thirdly, the findings are not inconsistent with either peripheral effects on the inner ear and eye or accompanying effects on the central nervous system (20).

The findings in this study are limited by the retrospective design, low subject numbers, and concerns about how to generalize the data beyond the Havana diplomatic community. However, despite these limitations and the absence of a detailed neuro-ophthalmologic consultation and exam, it remains uncertain whether the changes in pupil responses during the binocular disparity vergence tasks reflect direct ocular effects, effects on visual pathways, or adaptive responses to perturbations yet to be identified. Because the binocular disparity stimuli were predictable and relatively brief, one cannot exclude the possibility of a contribution of an impaired executive function to performance on these tasks, given the elevated prevalence of abnormal antisaccade task error rates in both subjects with acute mTBI (21) and the Havana affected cohort (2) Randomized disparity testing may be helpful in resolving the latter issue in the future. Nevertheless, our data demonstrate that the behavior of the Havana affected subjects in these tasks differed markedly from the convergence eye and the pupil coordination pattern in the acute mTBI subjects. These differences in objective performance need to be considered in the management of individuals showing features of the Havana affected group. For example, traditional mTBI treatment approaches that target convergence training may not be effective in this distinct patient population.

## Conclusion

In late 2016, diplomats in Havana, Cuba, began presenting with a unique symptom complex after perceiving a strange noise and/or feeling a pressure field in their domicile. There have been previous reports on both the initial as well as the longer-term findings in this population. This is the first report that examines the function of these individuals on a test that examined binocular disparity-driven eye and pupil movements during the acute time period after exposure. The patterns of response in these individuals are markedly different than those seen in a group of individuals with usual acute mild traumatic brain injury and from controls with no injury. The results from these tests permitted an objective discrimination of the groups with >91% accuracy and no confusion between the Havana subjects and the subjects with acute mild traumatic brain injury. This pattern difference may be a useful screen for individuals who report a similar exposure pattern. Furthermore, their distinctive presentation may help guide in treatment decisions to address the mechanisms that contribute to their unusual symptom complexes. At the current time, however, this remains an empirical observation and more work is needed to study the findings.

## Data Availability Statement

The datasets generated for this study are available on request to the corresponding author.

## Ethics Statement

The studies involving human participants were reviewed and approved by The Institutional Review Boards (IRBs) at the University of Miami and Madigan Army Medical Center approved the prospective studies of control subjects and subjects with mTBI. The IRB at the the University of Miami approved the retrospective study of clinical data from the Havana Affected cohort. The University of Pittsburgh IRB approved the analysis of de-identified data from all groups. The patients/participants provided their written informed consent to participate in this study.

## Author Contributions

CB, MS, BL, and MH contributed to the conception and design of the study and contributed to manuscript revision. CB performed the data analysis and wrote the first draft of the manuscript. CB, BL, and MH wrote sections of the manuscript. AK contributed only to device design, modifications and to device-related technical aspects of the manuscript. All authors read and approved the submitted version.

## Conflict of Interest

The authors declare that the research was conducted in the absence of any commercial or financial relationships that could be construed as a potential conflict of interest. The reviewer TA declared a past collaboration with one of the authors MH to the handling editor.

## Figures and Tables

**Figure A1:**
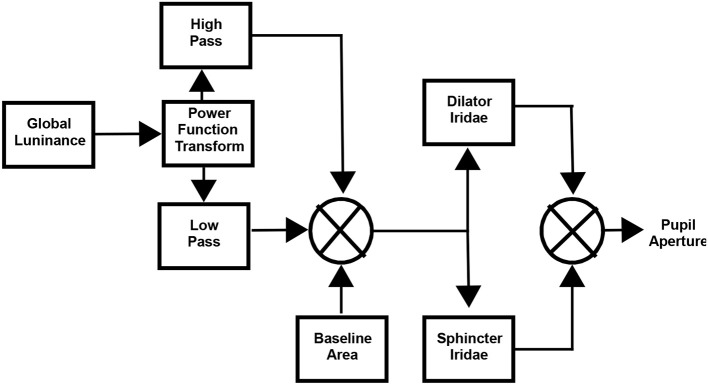
Schematic diagram of the model for dynamic analysis of pupillary light response data. This model is a lumped representation of the processes described in a series of papers from the Stark laboratory, spanning several decades (8, 10–13).

## Appendix

The model for analysis of the pupillary light responses, based upon the work of Stark and collaborators (8, 10–13), is summarized in Figure A1. The global luminance was transformed through a power function, where *b* is a fractional exponent that mimics the neural and the perceptual encoding of intensity (22, 23); initial values for model estimation procedures (below) were set by linear regression estimation of the relationship between the logarithms of the depth of each pupillary response and the logarithm of the stimulus intensity for that response. The luminance signals were then processed in parallel by a high-pass mechanism with transfer function $\frac{{\text{K}}_{\text{lh}}}{\;}\text{s }0.085s+1$ and a low-pass mechanism with transfer function $\frac{{\text{K}}_{\text{ll}}}{\;}0.15s+1$ (where “s” is a variable for the Laplace representation of the transfer function). The sum of these signals drives the iris musculature, with faster dynamics and a shorter delay for the sphincter muscle ($\frac{{\text{K}}_{\text{s}}{\text{e}}^{-0.2\text{s}}}{0.35+1}$) than the dilator muscle ($\frac{K_{d}e^{-0.5s}}{3.5s+1}$). The difference in the dynamic properties of these muscle is based upon published findings from bovine muscle (24). Non-linear least squares regression (“lsqnonlin.m” function in MATLAB) was used to estimate the power function and the gain parameters from experimental data. The models were fitted initially for each eye. Since there was no significant difference between the left and the right pupil responses for these binocular stimuli, the responses were then averaged. The parameter estimates in the results section are from the averaged data.
